# TREM2 brain transcript-specific studies in AD and TREM2 mutation carriers

**DOI:** 10.1186/s13024-019-0319-3

**Published:** 2019-05-08

**Authors:** Jorge L. Del-Aguila, Bruno A. Benitez, Zeran Li, Umber Dube, Kathie A. Mihindukulasuriya, John P. Budde, Fabiana H. G. Farias, Maria Victoria Fernández, Laura Ibanez, Shan Jiang, Richard J. Perrin, Nigel J. Cairns, John C. Morris, Oscar Harari, Carlos Cruchaga

**Affiliations:** 1 Department of Psychiatry, Washington University School of Medicine, St. Louis, MO USA; 2 NeuroGenomics and Informatics, Washington University School of Medicine, St. Louis, MO USA; 3 Hope Center for Neurological Disorders, Washington University School of Medicine, St. Louis, MO USA; 4 Knight Alzheimer’s Disease Research Center, Washington University School of Medicine, St. Louis, MO USA; 5 Department of Neurology, Washington University School of Medicine, St. Louis, MO USA; 6 Department of Pathology and Immunology, Washington University School of Medicine, St. Louis, MO USA

**Keywords:** TREM2, RNAseq, Soluble TREM2, R47H, Alzheimer’s disease, Brain transcripts, Risk variants

## Abstract

**Background:**

Low frequency coding variants in *TREM2* are associated with Alzheimer disease (AD) risk and cerebrospinal fluid (CSF) TREM2 protein levels are different between AD cases and controls. Similarly, TREM2 risk variant carriers also exhibit differential CSF TREM2 levels. *TREM2* has three different alternative transcripts, but most of the functional studies only model the longest transcript. No studies have analyzed *TREM2* expression levels or alternative splicing in brains from AD and cognitively normal individuals. We wanted to determine whether there was differential expression of *TREM2* in sporadic-AD cases versus AD-*TREM2* carriers vs sex- and aged-matched normal controls; and if this differential expression was due to a particular *TREM2* transcript.

**Methods:**

We analyzed RNA-Seq data from parietal lobe brain tissue from AD cases with *TREM2* variants (*n* = 33), AD cases (*n* = 195) and healthy controls (*n* = 118), from three independent datasets using Kallisto and the R package tximport to determine the read count for each transcript and quantified transcript abundance as transcripts per million.

**Results:**

The three *TREM2* transcripts were expressed in brain cortex in the three datasets. We demonstrate for the first time that the transcript that lacks the transmembrane domain and encodes a soluble form of TREM2 (sTREM2) has an expression level around 60% of the canonical transcript, suggesting that around 25% of the sTREM2 protein levels could be explained by this transcript. We did not observe a difference in the overall *TREM2* expression level between cases and controls. However, the isoform which lacks the 5′ exon, but includes the transmembrane domain, was significantly lower in *TREM2*- p.R62H carriers than in AD cases (*p* = 0.007).

**Conclusion:**

Using bulk RNA-Seq data from three different cohorts, we were able to quantify the expression level of the three *TREM2 *transcripts, demonstrating: (1) all three transcripts of them are highly expressed in the human cortex, (2) that up to 25% of the sTREM2 may be due to the expression of a specific isoform and not TREM2 cleavage; and (3) that *TREM2* risk variants do not affect expression levels, suggesting that the effect of the *TREM2* variants on CSF levels occurs at post-transcriptional level.

**Electronic supplementary material:**

The online version of this article (10.1186/s13024-019-0319-3) contains supplementary material, which is available to authorized users.

## Introduction

The Triggering Receptor Expressed in Myeloid cells 2 (TREM2) is a type 1 transmembrane receptor protein expressed on myeloid cells including microglia, monocyte-derived dendritic cells, osteoclasts and bone-marrow derived macrophages [1, 2]. TREM2 possesses an immunoglobulin-like extracellular domain, a transmembrane region and a short cytoplasmatic tail. In the brain, it is primarily expressed by microglia and has been shown to control two signaling pathways: regulation of phagocytosis and suppression of inflammatory reactivity [3, 4]. In the case of phagocytosis, there is a very strong relationship between TREM2 and the adaptor protein DAP12, also called TYROBP [5]. Homozygous loss-of-function mutations in *TREM2* or *DAP12* cause a rare and fatal disease known as Nasu-Hakola disease (NHD) or polycystic lipomembranous osteodysplasia with sclerosing leukoencephalopathy (PLOSL) which is characterized by an early-onset frontotemporal dementia-like phenotype and bone cysts [6, 7]. One of the leading hypotheses to explain the pathology associated with NHD is that lack of activity of *TREM2* or *DAP12* causes microglia inactivation and the accumulation of necrotic debris from apoptotic neurons [8].

This relationship with NHD prompted an effort to identify allelic variants in the *TREM2* coding region that could also confer risk to Alzheimer’s disease. In 2013, several studies found that heterozygous expression of *TREM2* p.R47H [9–24] and p.D87N [15] variants were significantly associated with AD risk. Other variants associated with the risk of AD include p.D87N [13, 15], p.R62H [17, 24], p.L211P, p.T96K and p.H157Y [16, 17]. Among all these variants, p.R47H was validated in neuropathological-confirmed cases [25] and was shown to increase the risk for late onset AD to a similar extent as the ApoE ɛ4 allele [15, 18]. However, the functional impact of the variant is not completely understood. In in-vitro studies, p.R47H has been shown to reduce the binding of Aβ oligomers, APOE and phosphatidylserine due to structural changes in TREM2 [26–29]. In in-vivo studies, the expression of human p.R47H in *Trem2* knockout mice suggested that the mutation confers a loss-of-function phenotype [30]. In 2018, two independent studies [31, 32] generated *Trem2* R47H knock-in mice using CRISPR/Cas 9 technology. Both mice showed reduced *Trem2* mRNA and protein levels, however the study from Xinag et al. [32] found that *TREM2* mRNA level in IPSC-derived human microglia-like cells and in patients’ brains with p.R47H were normal.

Most of *TREM2* AD-risk variants are located in exon 2, which codes for the Ig-like V type domain, suggesting a possible modification in the interaction between TREM2 and its ligands. Only two other risk variants associated to AD are located in different exons, p.H157Y in exon 3 and p.L211P in exon 4 of the transcript that encodes the soluble form of *TREM2* [15]. Kober et al. [33], presents an interesting hypothesis regarding loss of function in *TREM2*. In NHD, the TREM2 protein is not expressed, or it is expressed in a misfolded form that does not appear in the membrane; either scenario leads to a complete loss of function and a severe early-onset dementia. In AD, the risk variants are expressed in the cell membrane, but their binding capacity is lower than that of the WT, leading to a partial loss of function causing a less severe late-onset dementia. This hypothesis could explain why heterozygous NHD variants, including p.Q33X, p.Y38C, and p.T66 M, have been found in rare AD cases in heterozygous sate [15].

In 2014, it was reported that there are at least three *TREM2* transcripts that are expressed in human brain [17]. The first isoform, ENST00000373113, is the canonical and the longest *TREM2* transcript which consists of five exons. This transcript has a transmembrane domain, and it is the transcript that is normally modeled in functional studies. The second isoform, ENST00000373122, lacks exon 5, is the second longest transcript, and also includes the transmembrane domain. Both isoforms are anchored to the cell membrane due to their transmembrane domain, and both isoforms can undergo a sequential proteolytic processing by disintegrin and metalloproteinase domain-containing protein (ADAM) family, including ADAM10 and ADAM17, leading to the shedding of the ectodomain and producing a soluble TREM2 [34, 35]. The third isoform, ENST00000338469, is the shortest with an alternative spliced isoform that excludes exon 4. This isoform most likely encodes a soluble form of *TREM2* (*sTREM2*) due to the lack of exon 4 which encodes the transmembrane domain of the receptor.

A recent study [36] has shown the relevance of cerebrospinal fluid (CSF) *sTREM2* as a biomarker for AD progression due to its elevation in AD patients. It is unknown whether the sTREM2 is only the cleavage product of the cell-surface expressed protein or also the expression of the shortest soluble form.

The goal of this study is to quantify the expression level of the three *TREM2* transcripts in AD and control brains, to determine whether there is differential expression of *TREM2* in the three groups, sporadic-AD cases (cases), AD-TREM2 (TREM2-carriers) carriers and controls (controls) and finally to determine whether these differences are due to the differential expression of a particular *TREM2* isoform.

## Materials and methods

### Subjects and samples

The number of participants is presented in Table 1; they were grouped into three cohorts: Washington University in St. Louis Knight-ADRC Brain Bank (51 participants), MSBB-BM36, (132 participants) and MCBB (162 participants) all of whom were European-Americans. Briefly, the Knight-ADRC cohort included 15 participants with late-onset AD (NIA-AA criteria: intermediate or high) [37] with known risk variants in *TREM2* gene (Table 2), 12 non-demented controls and 39 sporadic late-onset AD cases. Because the variants p.D87N and p.R136W were associated with NHD and likely lead to neurodegeneration through a different mechanism from that of the AD risk variants, they were not used in the meta-analysis for TREM2 carriers. For MSBB-BM36, the number of non-demented control and sporadic late-onset AD cases were 28 and 93, respectively, and a total of 11 AD cases carried a TREM2 risk variant (see Table 2). In the case of MSBB-BM36, there were 78 non-demented controls, 77 sporadic late-onset AD, and 7 AD cases with a TREM2 risk variant (see Table 2). The RNA quality for samples in the study was good, with an average RNA integrity (RIN) number over five in all the cohorts. The age of death was also consistent in all cohorts, with a mean over 80 years old.

**Table 1:** Demographic Characteristics of study participants

	Controls	Alzheimer’s Disease Sporadic (Cases)	TREM2-carriers
Knight-ADRC			
No. of patients	12	24	15
Gender (% male)	33.3	45.8	53.3
Mean age at death (SD), years	90.1 (8.9)	85.1 (8.6)	84.5 (6.3)
Mean RIN (SD)	6.7 (1.2)	5.9 (1.4))	6.8 (1.3)
APOE genotype (ε4+)%	8.3	45.8	53.3
Mount Sinai Brain Bank (MSBB-BM36)			
No. of patients	28	93	11
Gender (% male)	50	32.3	45.5
Mean age at death (SD), years	80.9 (9.1)	84.4 (7.2)	84.3 (4.6)
Mean RIN (SD)	6.6 (1.1)	5.9 (1.5)	6.0 (1.7)
APOE genotype (ε4+)%	11.8	60	50
Mayo Clinic Brain Bank (MCBB)			
No. of patients	78	77	7
Gender (% male)	51.3	42.9	14.3
Mean age at death (SD), years	82.5 (8.9)	82.3 (7.8)	81.6 (4.6)
Mean RIN (SD)	7.6 (1.0)	8.6 (0.6)	8.3 (0.3)
APOE genotype (ε4+)%	10.3	51.9	57.1

**Table 2:** Number of subjects with the TRME2 variants in each study

TREM2 Variants	Knight-ADRC	Mount Sinai Brain Bank (MSBB-BM36)	Mayo Clinic Brain Bank (MCBB)
p.D87N^a^	1	0	0
p.G219C	0	0	0
p.R62C	0	1	0
p.E151K	0	0	0
p.H157Y^a^	1	0	0
p.L211P_T96K	1	0	0
p.R136W^a^	1	0	0
p.R52H	1	0	0
p.L133 L	0	1	1
p.R47H	4	6	1
p.R62H	8	3	5

^a^ Variants associated to NHD phenotype, when present on homozigous state

L211P_T96K: These two variants are in LD and are analyzed as a group

### Washington University in St. Louis knight-ADRC brain bank

Brain tissue was provided by the Washington University in St. Louis Charles F. and Joanne Knight Alzheimer’s Disease Research Center Brain Bank (Knight ADRC), all cases were recruited as research participants and underwent a standard battery of tests [38]. Clinical Dementia Rating (CDR) scores were obtained and the estimated CDR at the time of death was determined by telephone interview [39]. Neuropathological assessment was undertaken by NJC and RJP, with each case assessed using the NIA-AA neuropathologic diagnostic criteria. Additional postmortem data including post-mortem interval and brain weight were also available [40].

From these clinically and neuropathologically well-characterized cases [41], one to two grams of frozen (−80C) parietal lobe tissue (inferior parietal lobe) was dissected and made available for this study. For each case, post-mortem consent for a brain-only autopsy brain was obtained and was approved by Washington University in St. Louis institutional review board. Briefly, RNA was extracted from frozen brain tissues using the Tissue Lyser LT and RNeasy Mini Kits (Qiagen, Hilden, Germany) following the manufacturer’s instructions. RIN and DV200 were measured with the RNA 6000 Pico Assay on the Bioanalyzer 2100 (Agilent Technologies). The RIN is determined by the Bioanalyzer, taking into account the entire electrophoretic trace of the RNA including the presence or absence of degradation products. All the RIN values were acceptable for further analysis. The DV200 value is defined as the % of nucleotides greater than 200 nt. Each sample yield was determined by the Quant-iT RNA Assay (Life Technologies) on the Qubit Fluorometer (Fisher Scientific). The cDNA libraries were prepared with the TruSeq Stranded Total RNA Sample Prep with Ribo-Zero Gold kits (Illumina) and then sequenced by HiSeq 4000 (Illumina) at the McDonnell Genome Institute, Washington University in St. Louis. RNA-seq paired end reads with a read length of 2 × 150 bp were generated using Illumina HiSeq 4000 with a mean coverage of 80 million reads per sample.

### Mayo Clinic brain Bank (MCBB)

Mayo Clinic Brain Bank RNA-seq data was downloaded from the AMP-AD portal (synapse ID = 5,550,404; accessed January 2017). Paired end reads of 2 × 101 base pairs were generated by the Illumina HiSeq 2000 sequencer, for an average of 134.9 million reads per sample. RNA-seq based transcriptome data was generated from post-mortem brain tissue collected from the cerebellum (273 samples) and temporal cortex (275 samples) of Caucasian subjects. For this study, the temporal cortex samples were chosen. RNA was extracted using Trizol® and cleaned with the Qiagen RNeasy kit. RIN measurements were performed with Agilent Technologies 2100 Bioanalyzer. Libraries were prepared by the Mayo Clinic Medical Genome Facility Gene Expression and Sequencing Cores with the TruSeq RNA Sample Prep Kit (Illumina).

### Mount Sinai brain Bank (MSBB)

Mount Sinai Brain Bank RNA-seq data was downloaded from the AMP-AD portal (synapse ID = 3,157,743; accessed January 2017). Single end reads of 100 nucleotides were generated by the Illumina HiSeq 2500 System (Illumina, San Diego, CA), for an average of 38.7 million reads per sample. (Bank, 2016 #3723). It contains 1030 samples collected from four post-mortem brain regions of 300 subjects: the anterior prefrontal cortex (BM10), the superior temporal gyrus (BM22), parahippocampal gyrus (BM36), and the inferior frontal gyrus (BM44). RNA-seq was generated using the TruSeq RNA Sample Preparation Kit v2 and Ribo-Zero rRNA removal kit (Illumina, San Diego, CA) [https://www.synapse.org/#!Synapse:syn3157743]. The parahippocampal gyrus (BM36) is a cortical region in the medial temporal lobe that projects to surrounds the hippocampus and plays an important role in both spatial memory [42] and navigation [43, 44]. For this reason, BM36 was selected for further analysis for this study.

### TREM2 variant calling

For Knight ADRC, *TREM2* was sequenced using pooled-DNA sequencing designed as described previously [45]. All polymorphisms were validated by Sequenom and KASPar genotype for each individual included in the pool. For Mayo and the Mount Sinai datasets, *TREM2* variant calling was performed separately for 30X WGS as described before [46–48], following GATK’s 3.6 Best Practices (https://www.broadinstitute.org/gatk/) Variants were called in a region defined by the capture targets of the Agilent SureSelect Human All Exon V5 Kit, plus 100 bp of padding added to each capture target end. WGS data was filtered to remove low complexity regions, and regions with excessive depth. Only those variants that scored above the 99.5% confidence threshold were considered for analysis; additional variant filters included allele-balance (AB = 0.3–0.7). We also performed variant calling in the RNA-seq to determine if the TREM2 variants were expressed. We confirm that in all cases the alternative allele is expressed validating the genotypes for all individuals.

### RNA-seq expression quantification

FastQC [49] was used to assess sequencing quality. The RNA-seq was aligned to the human GRCh37 primary assembly using STAR (ver 2.5.2b). Read alignments were further evaluated by using PICARD CollectRnaSeqMetrics (ver 2.8.2) to examine read distribution across the genome. We employed Kallisto (v0.42.5) [50] and tximport [51] to determine the read count for each transcript and quantified transcript abundance as transcripts per kilobase per million reads mapped (TPM), using gene annotation of *Homo sapiens* reference genome (GENCODE GRCh37) for each participant from Knight-ADRC, MCB and MSBB-BM36 independently, with the following parameters: -t 10 -b 100. Then we summed the read counts and TPM of all alternative splicing transcripts of a gene to obtain gene expression levels. Due to the positive skewness of TPM values, we calculate their logarithm10 (log10TPM) for further analysis.

### Immunoprecipitation

Immunoprecipitation was performed as previously described [52]. In brief,  brain tissue were lysed in T-PER buffer (Thermo Scientific) and 1X Protease Inhibitor Cocktail (Sigma-Aldrich). Brain homogenate were precleared for 1 h at 4 °C with 20 uL of Pierce™ Protein G Agarose (Thermo Scientific). TREM2 protein was immunoprecipitated overnight at 4 °C with 5 μg of purified mouse monoclonal anti-TREM2 antibodies directed against the extra-cellular portion of human TREM2 protein (clone 20G2 and 29E3). 20 μL of Protein G agarose were added to the antigen-antibody complex and incubated for 2 h at room temperature. Precipitates were washed four times in cold phosphate buffer saline (PBS) with protease inhibitors (Sigma-Aldrich). SDS gel-loading buffer was added to the complex-bound resin, incubated for 5 min at 95 °C, separated by SDS–PAGE, transferred to PVDF membranes (BIO-RAD) and probed with the mouse anti-TREM-2 mAbs supernatants (clones 10B11 and 21E10). Densitometric semi-quantification was performed using ImageJ software (National Institutes of Health) [53].

### Statistical analysis

We employed a linear regression model to test the association between the log10TPM and each comparison group (R Foundation for Statistical Computing, ver.3.3.3) for the Knight-ADRC, MCBB and MSBB (BM36) cohorts. RIN number, age at death and gender were used as covariates for each analysis. All studies showed different absolute log10TPM values, due to the different library preparation or brain region. Therefore, for the meta-analysis, we used Stouffer’s Z-score method, which is based on Z-scores rather than *p*-values, allowing incorporation of study weights based on the sample size for each cohort. The definition of statistical significance was nominal *P*-value (*p* < 0.05) and the same direction of effects (that is, the sign of β) in each study to avoid discounting true positive associations.

## Results

### All three *TREM2* transcripts are highly expressed in human cortex

**Fig. 1:**
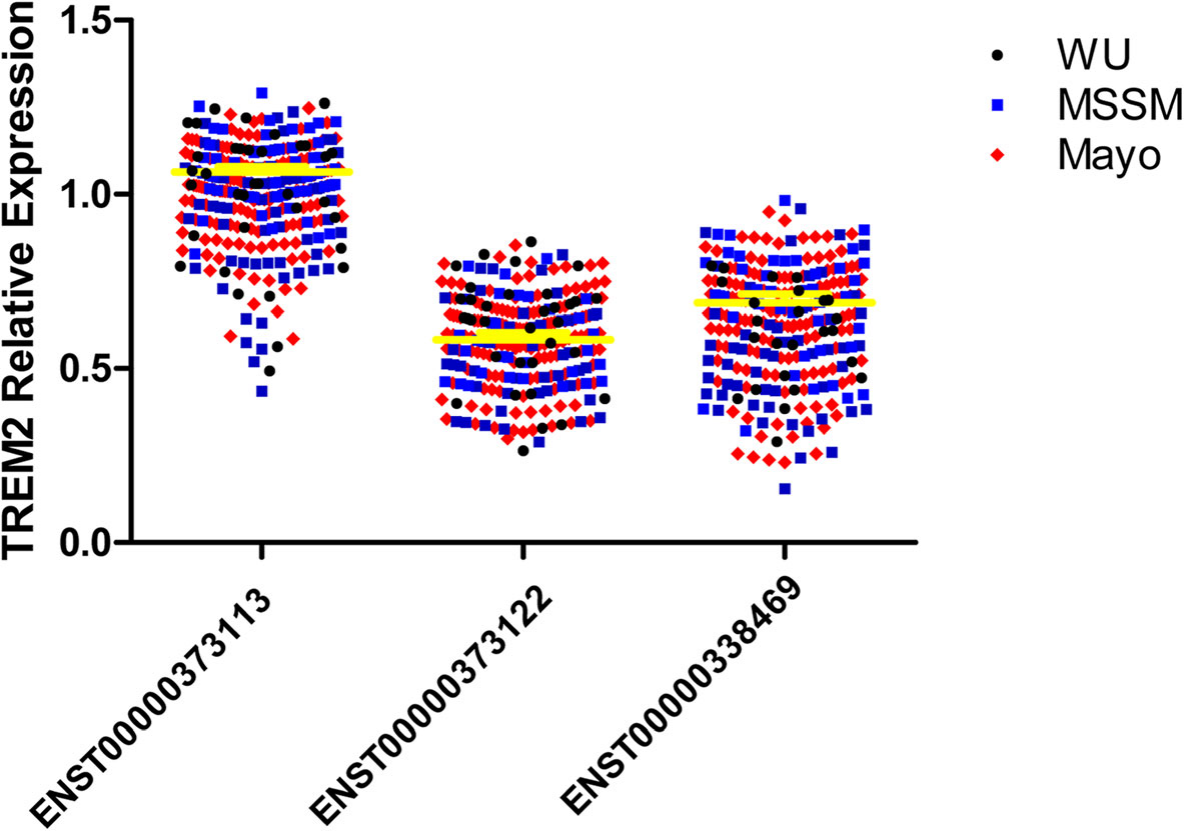
Relative quantification of the expression of each isoform in Alzheimer’s disease cases, controls and *TREM2* risk variant carriers. Total log10 RNA count was calculated using Kallisto and the results were expressed relative to the log10RNA counts of the common transcript ENST00000373113. There is a clear overlapping distribution for each of the studies, the yellow line represents the mean overall value for each transcript among the different studies. There were different expression counts among the different transcripts (see Table 5)

We first wanted to determine whether *TREM2* transcripts are expressed in the brain cortex, then to quantify each transcript to determine their relative abundance and possible importance. In order to be able to combine and compare expression levels across datasets, we normalized the log10TPM using the mean expression of the longest transcript as a reference. Log10TPM counts and analyses results can be found in the Additional file (Additional file 1: Table S1). We found that the canonical *TREM2* (ENST00000373113) transcript has significantly higher expression than the other two transcripts (*p* = 1.13 × 10^− 120^ and 4.76 × 10^− 107^, respectively, Fig. 1, Tables 3 and 4). The transcript that encodes the soluble TREM2 isoform (ENST00000338469) had the second highest expression level, and it is expressed at 62% of the canonical transcript. The transcript with the lowest expression level is the transcript that encodes a shorted transmembrane protein (ENST00000373122) and is expressed at 58% of the level of the canonical transcript. This transcript also showed a significantly lower level of expression than the transcript that encodes the soluble form (*p* = 0.0003).

**Table 3:** Relative quantification of the expression of each isoform with ENST00000373113 as control

Study	Conditions	ENST00000373113 (canonical transcript)	ENST00000373122	ENST00000338469 (soluble TREM2)
All	Control + Case	1.00	0.58	0.62
	Control	0.97	0.56	0.62
	Case	1.02	0.58	0.63
Knight-ADRC	Control + Case	1.00	0.61	0.60
	Control	0.97	0.56	0.53
	Case	1.01	0.64	0.63
MSBB-BM36	Control + Case	1.00	0.56	0.61
	Control	0.94	0.55	0.60
	Case	1.02	0.56	0.61
MCBB	Control + Case	1.00	0.58	0.64
	Control	0.98	0.57	0.63
	Case	1.02	0.60	0.64

ENST00000373113 canonical transcript

ENST00000373122

ENST00000338469 soluble TREM2

**Table 4:** Wilcoxon test for unpaired two independent groups

Study	Conditions	ENST00000373113 vs ENST00000373122	ENST00000373113 vs ENST00000338469	ENST00000373122 vs ENST00000338469
All	Control + Case	1.13 × 10^− 120^	4.76 × 10^−107^	0.0002
	Control	1.57 × 10^−43^	2.61 × 10^−35^	0.0090
	Case	1.86 × 10^−79^	2.80 × 10^−74^	0.0095
Knight-ADRC	Control + Case	2.16 × 10^−12^	3.39 × 10^−11^	0.6423
	Control	6.66 × 10^−05^	0.0003	0.4789
	Case	1.07 × 10^−07^	1.58 × 10^− 07^	0.7850
MSBB-BM36	Control + Case	2.99 × 10e^−40^	1.95 × 10^−38^	0.0291
	Control	6.20 × 10^−08^	3.86 × 10^−07^	0.3969
	Case	1.86 × 10^−34^	2.79 × 10^− 33^	0.0475
MCBB	Control + Case	1.99 × 10^−72^	2.16 × 10^−61^	0.0003
	Control	1.04 × 10^−33^	3.43 × 10^− 26^	0.0051
	Case	9.33 × 10^−43^	9.98 × 10^− 39^	0.0238

Table shows the *p*-value for the test comparison among studies and each isoforms:

ENST00000373113 canonical transcript

ENST00000373122

ENST00000338469 soluble TREM2

We also performed similar analyses for stratified by case-control status, and for each dataset, separately. The expression levels were very consistent across datasets and independent of case-control status (Fig. 1, Tables 3 and 4). The relative expression of the transcript that encodes the soluble form (ENST00000338469) ranges from 53 to 65% of that of the canonical transcript (ENST00000373113) and for the ENST00000373122 transcript, expression ranges from 55 to 61%. This data confirms that the three isoforms are expressed and that does not correlate with case-control status, and that up to 25% of the extracellular sTREM2 could be due to the expression of the ENST00000338469 isoform and not a product of TREM2 cleavage.

### Correlation with protein levels

In order to determine if the transcript levels correlate with protein levels, we quantified TREM2 protein levels by Western Blot on 10 parietal brain samples from the Knight-ADRC that RNA-seq data was also available. We detected two bands (Additional file 1: Figure S1); the 50kD band correspond to the full length TREM2 [34]. The second band located ~30kD, which matches to the sTREM2 found in cerebrospinal fluid [52]. Then, we quantified the band corresponding to the sTREM2 band and analyzed if there was a correlation with the sTREM2 band with the different transcripts. We found a very strong correlation between the sTREM2 band vs the total mRNA *TREM2* levels (R^2^ = 0.77 *p* < 0.05). We also found that the sTREM2 band shown a correlation with the canonical isoform (R^2^ = 0.73 *p* < 0.05) and the isoform that codify for the soluble transcript (R^2^ = 0.42 *p* < 0.05), but not for the other transcript (R^2^ = -0.13 *p* > 0.05).

### TREM2 expression levels are not affected by disease status

Since CSF sTREM2 levels are significantly different in AD cases vs controls, our next step was to determine whether the overall *TREM2* gene expression and its transcript specific expression were associated with case-control status. For these analyses, TREM2 carriers were not included. The overall *TREM2* expression levels were higher in AD cases compared to controls, but this difference was not statistically significant (*p* = 0.11, Table 5). Similar results were found for the canonical transcript. However, we found a nominal association between the transcript that encodes *sTREM2*, in which AD cases have higher transcript levels (*p* = 0.04, Table 5). These results were consistent across datasets replicating this finding (Additional file 1: Tables S2-S4).

**Table 5:** TREM2 is similarly expressed in AD, controls and TREM2 risk variant carriers

	Overall *TREM2*	ENST00000373113 (canonical transcript)	ENST00000373122	ENST00000338469 (soluble TREM2)
Control vs Case	0.11	0.2	0.19	0.04^c^
Control vs TREM2-carriers	0.40	0.39	0.31	0.39
Case vsTREM2-carriers	0.11	0.26	0.19	0.15
Control vs p.R47H^a^	0.33	0.34	0.15	0.06
Case vs p.R47H^a^	0.24	0.38	0.35	0.11
Control vs p.R62H	0.41	0.48	0.06^b^	0.25
Case vs p.R62H	0.17	0.20	**0.007** ^**b**^	0.22

Table shows the p–value for the meta-analysis for Knight-ADRC, Mayo Clinic Brain Bank and Mount Sinai Brain Bank

Meta-analyses was performed using the Zscore Method

Statistical significant results with the same effect size direction are shown in red

^a^Data only available for Knight-ADRC and Mount Sinai Brain Bank

^b^Data only available for Knight-ADRC and Mayo Clinic Brain Bank

^c^Statistically significant results but with different effect size directions

### *TREM2* risk variants do not affect TREM2 expression levels or disease status

**Fig. 2:**
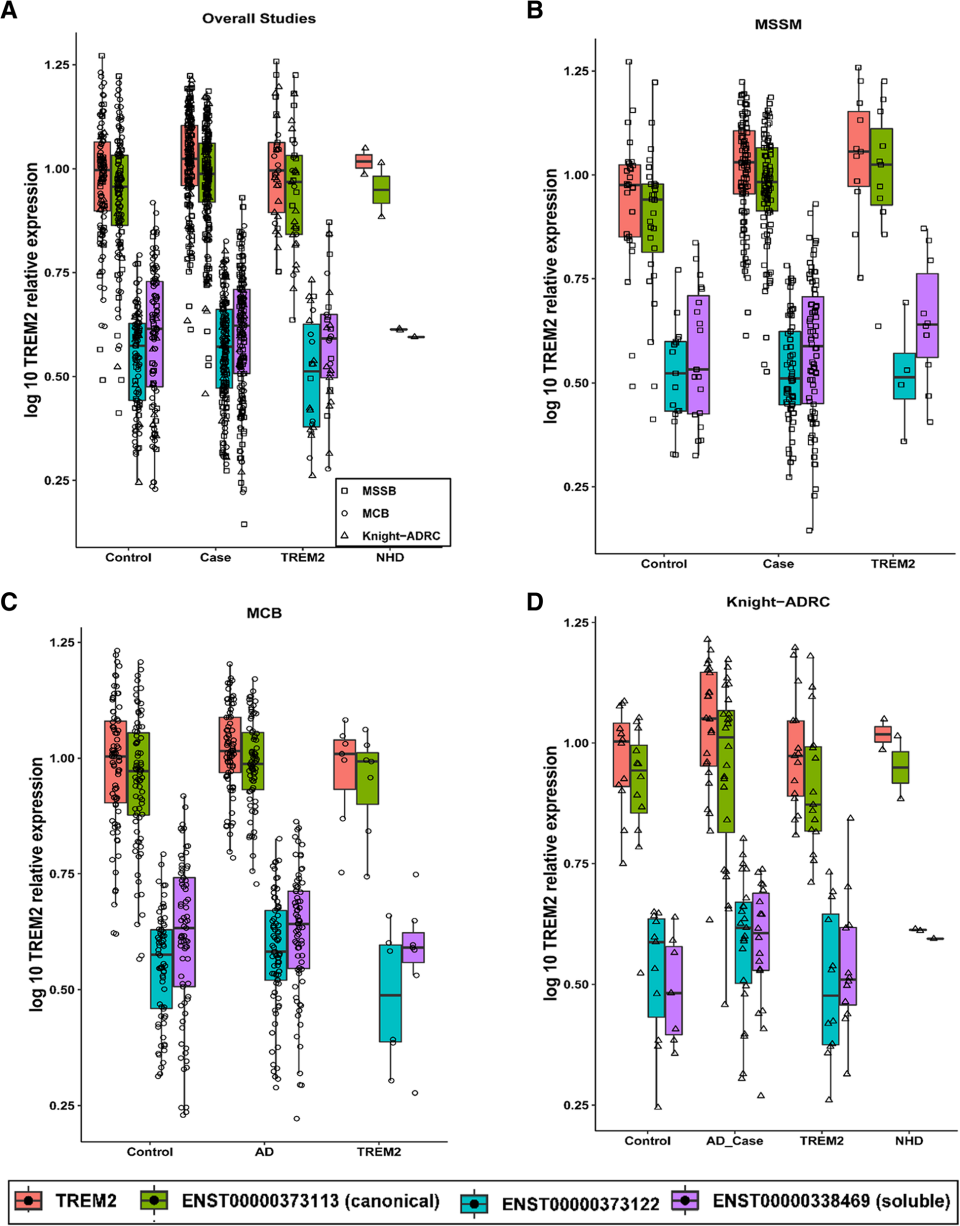
Overall *TREM2* and transcript-specific expression in Alzheimer’s disease cases, controls and *TREM2* risk variant carriers. Gene expression is showed as a log10 TPM values. **a** Overall, (**b**) Mount Sinai Brain Bank (MSBB) –BM36 study, (**c**) Mayo Clinic Brain Bank study and (**d**) Knight-ADRC study. Overall TREM2 refers to the total TREM2 expression of all the three transcripts

We also decided to analyze whether the *TREM2* risk variants (mainly p.R47H and p.R62H) were associated with TREM2 expression levels. We also included some variants that in homozygosity cause NHD, although in this study all the individuals with NHD variants are in a heterozygous state. NHD variants are loss-of-function variants that lead to very low TREM2 cell surface expression and low sTREM2 levels [36]. AD risk variants have also been postulated to be partial loss-of-function. Although, it is known that the loss-of-function for the NHD variants requires a post-transcriptional event, we wanted to confirm that these NHD or AD-risk variants have no effect at the mRNA level. We found that NHD variant-carriers have similar *TREM2* expression levels to AD cases, controls and other *TREM2* variants (Fig. 2). We also did not see any specific *TREM2* transcript effect for the NHD variants. All the transcripts were expressed at similar levels than in non-mutation carriers.

Then, we determined if the overall *TREM2* or specific transcript levels were significantly different between *TREM2* variant carriers (any *TREM2* variant) and cases or controls. We did not find any significant association, although TREM2-carriers showed nominally significant lower levels of the ENST00000373122 transcript (shorter, transmembrane protein) compared to controls (Fig. 2 and Table 5).

Finally, we performed specific analyses for the p.R47H and p.R62H variants. We did not find any significant difference in the overall or transcript-specific *TREM2* levels for the p.R47H variant. On the other hand, we found that the p.R62H variant carriers have significantly lower expression of the second transmembrane *TREM2* transcript (ENST00000373122) than cases (*p* = 0.007), and have lower expression compared to controls, although this difference is not statistically significant (Fig. 2 and Table 5). We did not find any difference for the other two transcripts or for the overall expression, suggesting that this effect is transcript specific.

## Discussion

Various studies suggest that *TREM2* and its variants contribute to the pathogenesis of AD [54], Parkinson’s Disease [55], and Amyotrophic Lateral Sclerosis risk [56]. However, it remains unclear if *TREM2* is a pro- or anti-inflammatory molecule [57]. For instance, in studies in macrophages or microglia, *TREM2* expression reduces the inflammatory response [4, 58–60], while in dendritic cells, *TREM2* expression exacerbates the inflammatory response [2, 61]. Due to these apparently conflicting results, a hypothesis was developed in which *TREM2* function depends on which cell type it is expressed. Therefore, if the cells are part of the innate immune system, *TREM2* acts as an anti-inflammatory molecule, but if they are part of the adaptive immune system, *TREM2* acts as a pro-inflammatory molecule. Most of the current research has been focused on *TREM2* gene expression levels and the *TREM2* variants associated with AD risk, however most of the studies have only focused on the canonical transcript, and not the activity or expression levels of the *TREM2* isoforms. It is also important to determine the expression levels of the three *TREM2* transcripts, as it is important to distinguish between the expression of the soluble *TREM2* isoform (ENST00000338469) and the s TREM2 produced by proteolytic cleavage. It is known that sTREM2 plays a role in AD pathogenesis [54] and can be used as biomarker for AD. Interestingly, the results from these studies shown that TREM2 levels are higher in AD individuals in CSF samples [36], which are consistent with our results at mRNA levels. However, one of the three *TREM2* transcripts expresses a soluble form of TREM2 that could be also be released extracellularly and be part of the overall pool of sTREM2 levels. Therefore, if we are to understand the role of sTREM2 in disease, we need to determine all the potential origins of this protein.

The expression of the three *TREM2* transcripts in brain was initially reported by Jin et.al [17]. In other studies, the expression of the canonical transcript (ENST00000373113) was the highest [62], followed by the transcript which encodes soluble *TREM2* (ENST00000338469) [63]. In this study, we used a large cohorts, totaling 345 samples to analyze not only the expression levels of the three *TREM2* transcripts but also to determine the association of AD case-control status and the *TREM2* risk variants with the expression levels.

We were able to detect and quantify the levels of three *TREM2* transcripts ENST00000373113, ENST00000373122 and ENST00000338469 using RNA-seq data from AD and control brains from three different, independent studies. Our analyses indicate that the canonical transcript (ENST00000373113) is expressed at twice the level of the other two other transcripts, and that this difference is very consistent across studies and between cases and controls. Even with this, our results indicate that the transcript that encodes sTREM2 represents the 25% of the total TREM2 mRNA suggesting that around 20–25% of the sTREM2 might be due to the expression of this transcript and not the cleavage of the cell membrane bound TREM2. In order to try to determine how much of the sTREM2 is produced by each transcript, we quantify total and sTREM2 from brain homogenates using Western-blots. As expected, we found a strong correlation of the sTREM2 band with the total TREM2 mRNA (R^2^ = 0.77 *p* < 0.05) and the canonical transcript (R^2^ = 0.73 *p* < 0.05). But we also found a strong and significant correlation with the transcript that codify for the soluble TREM2 transcript (R^2^ = 0.42 *p* < 0.05). This data supports that a proportion of the sTREM2 could be the results of the expression of the transcript that codify for the soluble isoform. However, there are several limitations to this experiment. Antibody-based detection assays are not able to distinguish between sTREM2 that was generated by proteolytic processing [35] or by alternative splicing [17]. Most of the epitopes used to generate anti-TREM2 antibodies are located in the extracellular portion of human TREM2 protein, which is shared by both the proteolytic and the transcribed forms [34, 35, 52, 64]. Therefore we are not able to determine the transcript of origin of the sTREM2 protein. The second limitation is that, because we quantified sTREM2 from brain homogenates, we can not distinguish between the extra and intra-cellular sTREM2 proteins. Therefore we cannot demonstrate that the sTREM2 protein produced by the soluble transcript is also present extracellularly. In addition, the TREM2 in the membrane is not all cleaved immediately. The actual proportion of sTREM2 that is produced by the direct expression of the ENST00000338469 transcript could be significantly higher or lower than 25%. More refined and specialized experimental techniques are needed to determine the origin of the soluble and extracellular presence of sTREM2.

In any case, recent studies indicate that minor changes in sTREM2 levels, of around 7–10% are enough to modulate AD risk [54, 65]. Therefore changes of expression levels by 25% should have a large impact on AD risk. Additional research to understand transcript specific *TREM2* regulation and determine the origin of sTREM2 are needed to fully understand the biology of TREM2.

Two previous papers [31, 32], that generated *Trem2* R47H knock-in mice using CRISPR/Cas 9 technology, showed reduced *Trem2* mRNA and protein levels, although the same studies suggest that these results were an artifact due to some unspecific events linked to the genome editing. It is also known that NHD variants are loss-of-function mutations. For instance, p.Y38C disrupts the correct disulfide formation, p.T66 M destabilizes the protein expression and p.V126G disrupts hydrophobic core of the protein. Furthermore, p.Y38 and p.V126 are conserved within the TREM family [33], implying that they are probably required to preserve the common fold within this family of receptors leading to lower sTREM2 levels, due to post-transcriptional events. On the other hand, variants p.R47H, p.R62H, p.N68K, p.D87N and p.T96K lie on the protein surface and are related to AD risk. Based on the location of these variants, we assumed that they would not affect surface expression and instead impact ligand binding [33]. Several studies indicate that CSF TREM2 levels are increased in AD risk variant p.R47H carriers, but in this case it is not clear if this is post-transcriptional, at the mRNA level. In our meta-analysis, the overall expression of *TREM2* at the gene level, and transcript-specific analyses in p.R47H or NHD carriers, *TREM2* and its isoforms were not significantly differentially expressed when compared to controls or AD cases, suggesting that the effects associated with the p.R47H variant are also at the post-transcriptional level. On the other hand, our data indicate the p.R62H carriers showed a significant level of the ENST0000037312, which also encodes a transmembrane TREM2 isoform. This finding could explain why TREM2 p.R62H carriers tend to have lower CSF TREM2 levels [36, 54].

## Conclusion

This study demonstrates that the three *TREM2* transcripts are highly expressed in human brains, and that the transcript that encodes the soluble form of *TREM2* might represent up to 25% of the soluble TREM2. Therefore, our results indicate that this transcript may play an important role in disease, and additional studies are needed to functionally characterize the rol of this transcript in disease. The implication of this transcript in AD pathogenesis is also supported by the presence of AD risk variant that are only present in the transcript that codifies for the soluble form. [17]. Thus in order to understand the biology of TREM2, it is important to perform functional studies and create cell and animal models that interrogate the three transcripts, not only the canonical transcript.

In this study, we were able to demonstrate the expression of the three transcripts of *TREM2* in postmortem human brain, including the soluble transcript that does not include a transmembrane domain. We also found the AD-risk variants influenced the expression of specific transcripts.

## Additional file

Additional file 1: **Table S1.** *TREM2* mean expression levels for each transcript and in each study. **Table S2.** Knight-ADRC results from each comparison group. **Table S3.** Mount Sinai Brain Bank (MSBB) –BM36 results from each comparison group. Table S4 Mayo Clinic Brain Bank (MCBB) results from each comparison group. **Figure S1.** Immuno precipitation sTREM2 in brain samples (DOCX 199 kb)

## Abbreviations

AD: Alzheimer’s disease
ADAM: A disintegrin and metalloproteinase domain-containing protein
BM10: Anterior prefrontal cortex
BM22: Superior temporal gyrus
BM36: Parahippocampal gyrus
BM44: Inferior frontal gyrus
CDR: Clinical Dementia Rating
CSF: Cerebrospinal fluid
DAP12: DNAX activation protein of 12 kDa
Knight-ADRC: Washington University in St. Louis Charles F. and Joanne Knight Alzheimer’s Disease Research Center Brain Bank
log10TPM: Logarithm10 TPM
MCBB: Mayo Clinic Brain Bank
MSBB: Mount Sinai Brain Bank
NHD: Nasu-Hakola Disease
PLOSL: Polycystic Lipomembranous Osteodysplasia with Sclerosing Leukoencephalopathy
sTREM2: Soluble form of TREM2
TPM: Transcripts per Million reads mapped
TREM2: Triggering Receptor Expressed on Myeloid cells 2
TYROBP: TYRO protein tyrosine kinase binding protein
