# Supplementary material for: TREM2 brain transcript-specific studies in AD and TREM2 mutation carriers
Source: Mol Neurodegener. 2019 May 8;14:18. doi: 10.1186/s13024-019-0319-3 (PMC6505298; doi:10.1186/s13024-019-0319-3)
Supplement: Supplementary file 1 — Table S1. TREM2 mean expression levels for each transcript and in each study. Table S2. Knight-ADRC results from each comparison group. Table S3. Mount Sinai Brain Bank (MSBB) –BM36 results from each comparison group. Table S4 Mayo Clinic Brain Bank (MCBB) results from each comparison group. Figure S1. Immuno precipitation sTREM2 in brain samples (DOCX 199 kb) [file 13024_2019_319_MOESM1_ESM.docx]

**Supplementary results**

**TREM2 brain transcript-specific studies in AD and TREM2 mutation carriers**

Jorge L Del-Aguila^a,b^, Bruno Benitez^a,b^, Zeran Li^a,b^, Umber Dube^a,b^, Kathie A. Mihindukulasuriya^a^, John P Budde^a,b^, Fabiana Farias^a,b^ , Maria Victoria Fernández^a,b^, Laura Ibañez^a,b^, Shan Jiang^a,b^, Richard J. Perrin ^b,c,e^, Nigel J. Cairns^b,c,d,e^ , John C Morris^c,d^ , Oscar Harari^a^, Carlos Cruchaga^a,b,c,*^

Affiliations:

*^a^Department of Psychiatry, Washington University School of Medicine, St. Louis, MO, USA*

^b^*Hope Center for Neurological Disorders, Washington University School of Medicine, St. Louis, MO, USA* 9

^c^*Knight Alzheimer’s Disease Research Center, Washington University School of Medicine, St. Louis, MO, USA;* 11

^d^*Department of Neurology, Washington University School of Medicine, St. Louis, MO, USA*

^e^*Department of Pathology and Immunology, Washington University School of Medicine, St. Louis, MO, USA*

* To whom correspondence should be addressed:

Carlos Cruchaga, PhD

Associate Professor

Department of Psychiatry

The Hope Center Program on Protein Aggregation and Neurodegeneration (HPAN)

Washington University, School of Medicine

425 S. Euclid Ave.

BJC Institute of Heath. Box 8134

St. Louis, MO 63110

Tel: 314-286-0546

Fax: 314-362-2244

email: ccruchaga@wustl.edu

*TREM2 expression levels are not affected by disease status*

Using the quasi-mapping mode of Kallisto, we were able to estimate the log10TPM for the *TREM2* gene level and each of its isoforms, for each of the studies and comparison groups (Supplemental Figure1). The mean expression patterns among the different studies and comparison groups are shown in Supplemental Table1. The mean expression values in the Knight-ADRC study for the *TREM2* gene level in the different groups were 1.87 (SD: 0.21), 1.98 (SD: 0.27), 1.89 (SD: 0.23) and 1.97 (SD: 0.09) for control, cases, carriers Trem2-carriers and NHD-carriers respectively. In the case of MSBB-BM36 study, the means expression values were 1.92 (SD: 0.33), 2.06 (SD: 0.26) and 2.13 (SD: 0.31) for control, cases, carriers TREM2-carriers. In the MCBB study, the mean expression values were 2.64 (SD: 0.37), 2.74 (SD: 0.25) and 2.60 (SD: 0.31). In the last two studies, no data was available for NHD carriers.

The log10TPM count for each transcript was expressed relative to the canonical transcript ENST00000373113 (Figure1, Table 4). The mean expression value of ENST00000373113 was almost twice as high as the transcript ENST00000373122 and the soluble TREM2 transcript ENST00000338469 when combined in all the studies, in controls (p= 1.57×10^-43^, p= 2.61×10^-35^), cases (p= 1.86×10^-79^, p= 2.80×10^-74^) and in control + cases (p= 1.13×10^-120^, p= 4.76×10^-107^). This pattern was observed in each independent study for each comparison group. The mean expression value of transcript ENST00000338469, which encodes soluble *TREM2*, was higher than ENST00000373122 when all studies were combined (control: p=0.0090, case: p= 0.0095 and control + case: p= 0.0002) most of the significance of these p values was driven by MCBB.

| **Supplementary Table 1:** ***TREM2* mean expression levels for each transcript and in each study** | | | | |
| --- | --- | --- | --- | --- |
| **Knight-ADRC** | | | | |
| **Transcript** | **Control**  **Mean (SD)** | **Cases**  **Mean (SD)** | **TREM2-carriers**  **Mean (SD)** | **NHD-carries**  **Mean (SD)** |
| TREM2 | 1.87 (0.21) | 1.98 (0.27) | 1.89 (0.23) | 1.97 (0.09) |
| ENST00000373113 | 1.74 (0.28) | 1.82 (0.37) | 1.76 (0.27) | 1.83 (0.18) |
| ENST00000373122 | 1.01 (0.27) | 1.14 (0.27) | 0.97 (0.30) | 1.18 (0.00) |
| ENST00000338469 | 0.95 (0.21) | 1.13 (0.25) | 1.04 (0.27) | 1.15(0.00) |
| **Mount Sinai Brain Bank-BM36** | | | | |
| TREM2 | 1.92 (0.33) | 2.06 (0.26) | 2.13(0.31) | NP |
| ENST00000373113 | 1.80 (0.36) | 1.97 (0.27) | 2.04 (0.34) | NP |
| ENST00000373122 | 1.06 (0.25) | 1.08 (0.25) | 1.06 (0.28) | NP |
| ENST00000338469 | 1.16 (0.33) | 1.18 (0.35) | 1.32 (0.31) | NP |
| **Mayo Clinic Brain Bank** | | | | |
| TREM2 | 2.64 (0.37) | 2.74 (0.25) | 2.60 (0.31) | NP |
| ENST00000373113 | 2.56 (0.38) | 2.65 (0.26) | 2.54 (0.26) | NP |
| ENST00000373122 | 1.48 (0.32) | 1.56 (0.34) | 1.31 (0.39) | NP |
| ENST00000338469 | 1.64 (0.46) | 1.66 (0.37) | 1.53 (0.40) | NP |
| Gene expression is showed in log10 Transcripts Per Million (log10TPM)  SD: Standard Deviation  NP. Not present | | | | |

| **Supplementary Table 2: Knight-ADRC results from each comparison group.** | | | | |
| --- | --- | --- | --- | --- |
|  | ***TREM2*** | **ENST00000373113**  **(canonical transcript)** | **ENST00000373122** | **ENST00000338469 (soluble TREM2)** |
| Control vs Case | 0.15/0.09 | 0.11/0.39 | 0.12/0.25 | **0.25/0.03** |
| Control vs  TREM2-carriers | 0.02/0.85 | 0.003/0.97 | -0.01/0.94 | -0.002/0.97 |
| Case vs  TREM2-carriers | -0.12/0.18 | -0.1/0.45 | **-0.23/0.03** | -0.07/0.47 |
| Control vs p.R47H | -0.01/0.96 | -0.09/0.69 | **-0.47/0.007** | 0.15/0.31 |
| Case vs p.R47H | -0.16/0.34 | -0.02/0.91 | -0.08/0.52 | -0.06/0.71 |
| Control vs p.R62H | -0.002/0.98 | -0.02/0.91 | -0.08/0.52 | -0.06/0.71 |
| Case vs p.R62H | -0.14/0.21 | -0.11/0.47 | -0.19/0.13 | -0.09/0.46 |

| **Supplementary Table 3: Mount Sinai Brain Bank (MSBB) –BM36 results from each comparison group.** | | | | |
| --- | --- | --- | --- | --- |
|  | ***TREM2*** | **ENST00000373113**  **(canonical transcript)** | **ENST00000373122** | **ENST00000338469 (soluble TREM2)** |
| Control vs Case | **0.16/0.001** | **0.19/0.001** | 0.05/0.45 | 0.10/0.18 |
| Control vs  TREM2-carriers | **0.22/0.03** | **0.25/0.02** | 0.03/0.84 | **0.25/0.04** |
| Case vs  TREM2-carriers | 0.06/0.34 | 0.06/0.38 | -0.02/0.83 | 0.15/0.11 |
| Control vs p.R47H | **0.26/0.03** | **0.27/0.05** | 0.03/0.87 | **0.29/0.04** |
| Case vs p.R47H | 0.09/0.28 | 0.06/0.52 | -0.003/0.98 | 0.19/0.13 |
| Control vs p.R62H | 0.23/0.17 | 0.33/0.09 | NA | 0.41/0.08 |
| Case vs p.R62H | 0.07/0.56 | 0.12/0.37 | NA | 0.25/0.23 |

| **Supplementary Table 4: Mayo Clinic Brain Bank (MCBB) results from each comparison group** | | | | |
| --- | --- | --- | --- | --- |
|  | ***TREM2*** | **ENST00000373113**  **(canonical transcript)** | **ENST00000373122** | **ENST00000338469 (soluble TREM2)** |
| Control vs Case | 0.005/0.93 | 0.004/0.95 | 0.07/0.23 | -0.10/0.19 |
| Control vs  TREM2-carriers | -0.07/0.63 | -0.05/0.73 | -0.17/0.23 | -0.17/0.37 |
| Case vs  TREM2-carriers | -0.14/0.17 | -0.12/0.25 | -0.27/0.07 | -0.13/0.36 |
| Control vs p.R62H | -0.14/0.43 | -0.11/0.53 | **-0.35/0.04** | -0.21/0.35 |
| Case vs p.R62H | -0.19/0.10 | -0.16/0.17 | **-0.44/0.01** | -0.16/0.34 |

Figure S1

***
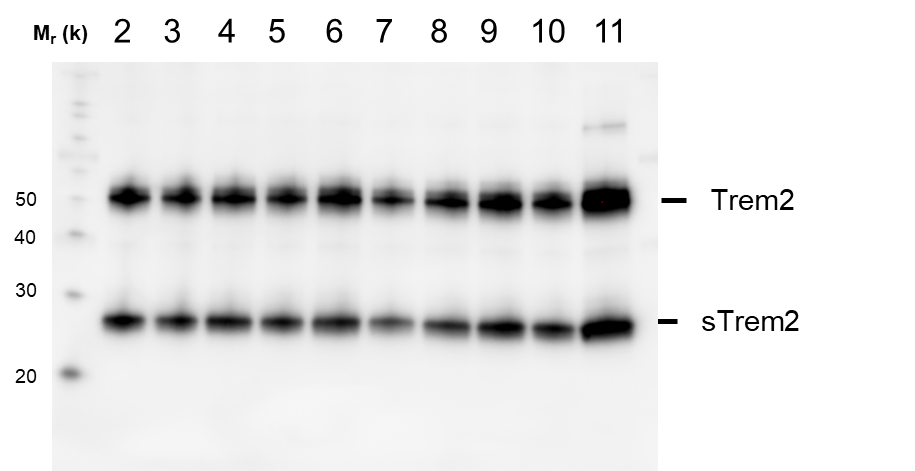
***

**Figure 1 Immuno precipitation sTREM2 in brain samples**
